# DNA Methylation Reorganization of Skeletal Muscle-Specific Genes in Response to Gestational Obesity

**DOI:** 10.3389/fphys.2020.00938

**Published:** 2020-07-31

**Authors:** Anna Prats-Puig, Sergi García-Retortillo, Miquel Puig-Parnau, Fidanka Vasileva, Raquel Font-Lladó, Sílvia Xargay-Torrent, Gemma Carreras-Badosa, Berta Mas-Parés, Judit Bassols, Abel López-Bermejo

**Affiliations:** ^1^University School of Health and Sport (EUSES), University of Girona, Girona, Spain; ^2^Complex Systems in Sport, National Institute of Physical Education and Sport of Catalonia (INEFC), Universitat de Barcelona (UB), Barcelona, Spain; ^3^Faculty of Physical Education, Sport and Health, Ss. Cyril and Methodius University, Skopje, North Macedonia; ^4^Pediatric Endocrinology, Girona Institute for Biomedical Research, Dr. Josep Trueta Hospital, Girona, Spain; ^5^Maternal & Fetal Metabolic Research, Girona Institute for Biomedical Research, Salt, Spain

**Keywords:** gestational obesity, skeletal muscle, umbilical cord tissue, methylation, network physiology

## Abstract

The goals were to investigate in umbilical cord tissue if gestational obesity: (1) was associated with changes in DNA methylation of skeletal muscle-specific genes; (2) could modulate the co-methylation interactions among these genes. Additionally, we assessed the associations between DNA methylation levels and infant’s variables at birth and at age 6. DNA methylation was measured in sixteen pregnant women [8-gestational obesity group; 8-control group] in umbilical cord using the Infinium Methylation EPIC Bead Chip microarray. Differentially methylated CpGs were identified with Beta Regression Models [false discovery rate (FDR) < 0.05 and an Odds Ratio > 1.5 or < 0.67]. DNA methylation interactions between CpGs of skeletal muscle-specific genes were studied using data from Pearson correlation matrices. In order to quantify the interactions within each network, the number of links was computed. This identification analysis reported 38 differential methylated CpGs within skeletal muscle-specific genes (comprising 4 categories: contractibility, structure, myokines, and myogenesis). Compared to control group, gestational obesity (1) promotes hypermethylation in highly methylated genes and hypomethylation in low methylated genes; (2) CpGs in regions close to transcription sites and with high CpG density are hypomethylated while regions distant to transcriptions sites and with low CpG density are hypermethylated; (3) diminishes the number of total interactions in the co-methylation network. Interestingly, the associations between infant’s fasting glucose at age 6 and *MYL6, MYH11, TNNT3, TPM2, CXCL2, and NCAM1* were still relevant after correcting for multiple testing. In conclusion, our study showed a complex interaction between gestational obesity and the epigenetic status of muscle-specific genes in umbilical cord tissue. Additionally, gestational obesity may alter the functional co-methylation connectivity of CpG within skeletal muscle-specific genes interactions, our results revealing an extensive reorganization of methylation in response to maternal overweight. Finally, changes in methylation levels of skeletal muscle specific genes may have persistent effects on the offspring of mothers with gestational obesity.

## Introduction

Excessive gestational weight gain is related to offspring obesity and related metabolic disorders, independently of pre-pregnancy body mass index (Wrotniak et al., 2008; Olson et al., 2009; Reynolds et al., 2010; Vickers, 2014). More importantly, the effects on the offspring persist into adolescence and adult life (Oken et al., 2008; Hochner et al., 2012).

Studies in rats showed that offspring born to mothers fed an obesogenic diet during pregnancy exhibit reduced skeletal muscle cross-sectional area and fiber number. These structural defects led to impaired muscle contractility (Bayol et al., 2005) and impaired insulin signaling pathway and mitochondrial function (Shelley et al., 2009). Skeletal muscle accounts for about 40% of the whole body mass and plays a central role in metabolic health (DeFronzo and Tripathy, 2009; Gardner and Rhodes, 2009) not only through the regulation of lipid and glucose metabolism (DeFronzo et al., 1981; Brüning et al., 1998) but also through the production of myokines (Pedersen, 2013; Karstoft and Pedersen, 2016). Defects in the formation of skeletal muscle *in utero* can lead to metabolic complications into adult life (Bayol et al., 2014) as there is no increase in muscle fiber numbers after birth (White et al., 2010).

In mammals, epigenetic regulation is crucial for a variety of different processes such as development, cell differentiation, and proliferation (Skinner, 2011). Numerous studies demonstrate that skeletal muscle can be programmed in part by epigenetic modifications (Jaenisch and Bird, 2003; Patel et al., 2014) and that changes in DNA methylome can be retained and accumulated over time (Jacobsen et al., 2012; Sharples et al., 2016a). Previous research have suggested that appropriate gene interactions, controlled by epigenetic modifications, are of key relevance to maintain cellular homoeostasis (Azuara et al., 2006). As pointed out by Bartsch et al. (2015) understanding the nature of such interactions, can provide useful information regarding the specific role of several physiological systems within an integrated network, during different physiological states (e.g., gestational obesity). However, the effect of gestational obesity on skeletal muscle-specific genes in humans, as well as the mechanisms underlying co-methylation network interactions (i.e., interaction among methylation patterns of individual CpG sites) within these genes in umbilical cord tissue, have been poorly studied (Akulenko and Helms, 2013; Sun and Sun, 2019). Thus, our goals were to examine in umbilical cord tissue if gestational obesity (1) was associated with changes in the DNA methylation of skeletal muscle-specific genes, (2) and could modulate the co-methylation interactions among these genes. Additionally, we also assessed the associations between methylation levels of skeletal muscle-specific genes in umbilical cord tissue and infant’s anthropometric and metabolic variables at birth and at age 6.

## Methods

### Study Population and Ethics

The study population included 16 apparently healthy pregnant Caucasian women delivering healthy infants. All pregnant woman had normal weight prior to pregnancy (18.5 < BMI < 24.9). The subjects were selected from a consecutively recruitment during the first trimester of pregnancy among those seen within a setting of prenatal primary care in l’Alt Empordà (Northeastern Spain). The selection of our samples was done to ensure the minimum variation between individual samples. Information on pregnancy, labor, and delivery characteristics was retrieved from standardized medical records. Women with major medical, surgical, or obstetrical complications, including multiple pregnancies, hypertension, gestational diabetes, or preeclampsia, and fetal growth restriction, malformations or asphyxia were excluded. Assisted reproductive technology (Lim et al., 2009) and smoking, drugs of abuse or alcohol addiction during pregnancy were also excluded.

The protocol was approved by the Institutional Review Board of Dr. Josep Trueta Hospital and informed written consent was obtained from all parents.

### Maternal Anthropometric Assessments

Maternal weight and height were assessed at each trimester during gestation and before delivery (between 37 and 41 weeks). Body-mass index (BMI) was calculated as weight divided by height squared (Kg/m^2^). We used maternal weight at the beginning of gestation as a proxy for pre-pregnancy weight. Maternal gestational weight gain was obtained as the difference between the last weight measurement before delivery and pre-pregnancy weight. We classified women into two groups: control group, in whom adequate weight gain during pregnancy (between 11.5 and 16kg) was evidenced, and gestational obesity group in whom excessive weight was gained during pregnancy (above 16 kg), as previously described by the Institute of Medicine guidelines (Olson, 2008).

### Infants Anthropometric and Metabolic Assessments

All infants were born at term pregnancy. After delivery, weight and length were measured using a calibrated scale and a measuring board. Gestational age- and sex-adjusted z-scores for birth weight and length were calculated using regional norms (Carrascosa et al., 2008). Ponderal index was calculated as follows: (birth weight in grams x 100) / (birth length in centimeters)^3^. From the children included at birth, those whose parents agreed to participate further in the study (*n* = 12) were followed-up at the age of 6 years. Their characteristics at birth did not differ from those who did not participate in the follow-up study. Weight was measured on a calibrated scale wearing light clothes, and height was measured with a Harpender stadiometer without shoes. BMI and age- and sex- adjusted z-scores were calculated as above. Fat mass percentage was assessed by bioelectric impedance (Hydra Bioimpedance Analyzer 4200; Xitron Technologies, San Diego CA, United States).

At birth, umbilical cord blood was collected from the vein immediately after birth and insulin was measured by immunochemiluminiscence (IMMULITE 2000, Diagnostic Products, Los Angeles, CA, United States). At 6 years of age, serum samples were obtained under fasting conditions and fasting glucose was analyzed by the hexokinase method.

### Umbilical Cord Tissue Sample Collection and DNA Methylation Analysis

Immediately after childbirth, a sample of umbilical cord tissue was collected and stored at −80°C. Genomic DNA was extracted from the Wharton’s jelly and blood vessels using the Gentra PureGene tissue kit (Qiagen). Sodium bisulfite conversion of DNA was performed, and the chemically modified DNA was then used to analyze the methylation status of over 850,000 individual CpGs in umbilical cord tissue using the Infinium Methylation EPIC Bead Chip microarray (Illumina) at IIS La Fe (Valencia, Spain). DNA methylation data quality control and normalization were performed using the minify R-package (version 1.26.2). Functional normalization and filters were applied to the raw data with the aim to discard probes with a detection *p* > 0.01, related to sexual chromosomes, within SNPs and multiple homologies. We also applied signal background subtraction and probes that lack signal values in one or more samples were also excluded. At the end, we obtained 27,262 probes with a *p* < 0.01. From those, 115 were related to skeletal muscle but only 38 were differently methylated between groups. Differentially methylated CpGs were identified with Beta Regression Models (false discovery rate (FDR) < 0.05 and an Odds Ratio (OR) > 1.5 or < 0.67). Each probe or CpG site on the array is annotated to a genomic location [Transcription starting site (TSS)1500, TSS200, 5’UTR, 1stExon, Body, and 3’UTR) and a location in the CpG islands [CpG islands, shores (<2 kb from the CpG island), shelves (2 to 4 kb from the CpG island), and open sea region (>4 kb from the CpG island)]. These were combined for each CpG site to form a unique genomic context annotation. According to each genomic location two groups were created: close (TSS1500, TSS200, 5’UTR, and 1stExon) and distant (Body and 3’UTR) to a transcription site.

Additionally, we used DNA methylation data from skeletal muscle biopsies from a publicly available sample set (EPIC series GSE114763; Seaborne et al., 2018) to test (1) the similarity between DNA methylation levels in umbilical cord tissue and skeletal muscle biopsies; (2) which genes can be modified by both, gestational obesity and resistance training.

### CpGs Co-methylation Matrices and Networks

We selected skeletal muscle-specific genes related to contractile function, structural features, myokines, and muscle myogenesis to create four categories of genes according to previous literature (Doherty et al., 2005; Griffin et al., 2010; Pedersen, 2013; Heissler and Sellers, 2014; Subbotina et al., 2015; Mukherjee et al., 2016; Munters et al., 2016; Gautel and Djinoviæ-Carugo, 2016; Cardinali et al., 2016; Chal and Pourquié, 2017; Dierck et al., 2017; Kim et al., 2018; Leal et al., 2018; Lin et al., 2018; Meng et al., 2019; Nieman and Pence, 2019; Pillon et al., 2020; Stavroulakis and George, 2020). To study the interaction among skeletal muscle-specific genes, the DNA methylation levels for the differentially methylated CpG site from umbilical cord tissue was used to construct one gene co-methylation matrix and one gene co-methylation network, for both control and gestational obesity groups. To create the co-methylation matrix (Figure 2A) we used the Pearson correlation coefficient to calculate the correlations of the methylation levels between every pair of individual CpGs sites. Then, to obtain the co-methylation network (Figure 2B) we only used the statistically significant correlations obtained in the co-methylation matrix (Ruan et al., 2010). With the aim of quantifying the interactions within each network (Bartsch et al., 2015) we also computed the number of links (i.e., number of significant correlations). Specifically, we calculated (a) the total number of links (Figure 2C) (b) the number of links among individual CpG sites within the same gene category (i.e., intra-category; contractibility, structure, myokines, and myogenesis; Figure 3A) and c) the number of links comparing categories pairwise (i.e., inter-category; contractibility vs. structure; contractibility vs. myokines; contractibility vs. myogenesis; structure vs. myokines; structure vs. myogenesis; myokines vs. myogenesis; Figure 3B). The links were divided into four types: strong positive links (SPL; Pearson coefficients > 0.8), intermediate positive links (IPL; 0.7 < Pearson coefficients < 0.8), intermediate negative links (INL; −0.7 > Pearson coefficients >−0.8), and strong negative links (SNL; Pearson coefficients <−0.8). Gene co-methylation matrices and networks were processed and obtained by means of Matlab R2016b (Mathworks, Natik, MA, United States). The visualization framework used in our results is based on previous studies analyzing network interactions among physiological systems during different physiological states (Bashan et al., 2012; Bartsch et al., 2015; Lin et al., 2020).

### Statistics

Differences in clinical variables and DNA methylation levels between groups (all, gene localization, location in CpG islands and genomic context) were examined by Mann-Whitney *U* test using SPSS 22.0 (SPSS Inc). The methylation change was calculated in relation to control group levels. The associations between methylation levels of skeletal muscle-specific genes in umbilical cord tissue and infant’s anthropometric and metabolic variables were analyzed by Spearman’s correlation. Significance level was set at *p* < 0.001 after multiple testing correction (0.05/38 comparisons).

## Results

Supplementary Table 1 shows the clinical variables in the mothers and their respective children at birth and at 6 years of age. We show that the two groups only differ in maternal weight gain during gestation and infant’s fasting glucose at 6 years of age.

### Gestational Obesity and DNA Methylation Changes

After the quality control of the methylation data we obtained 27,262 probes with a *p* < 0.01. From those, 115 were related to skeletal muscle (FDR <0.05) but only 38 were differently methylated between groups (FDR <0.05 and OR >1.5 or <0.67). Among the differently methylated individual CpG sites identified in umbilical cord tissue between mother with and without gestational obesity and according to only FDR criteria we found 6 CpGs related to contractile function, 65 related to structure, 28 CpG related to myokines and 16 related to myogenesis of skeletal muscle (Supplementary Table 2). From those, in Supplementary Table 3 we show the methylation levels for 38 individual CpG sites that showed differences in DNA methylation levels according to both, FDR and OR criteria, within the skeletal muscle-specific genes coding for contractile functions (*n* = 4), structure (*n* = 17), myokines (*n* = 12) and myogenesis (*n* = 5). For each identified gene, we report the known function and their defined role related to skeletal muscle (Supplementary Table 4). Our results showed that, except for gene CDH15-2 (structure), the effect of gestational obesity on DNA methylation might depend on initial methylation levels: compared to control group, gestational obesity promotes a hypermethylation effect in highly methylated genes (Supplementary Figure 1A and Supplementary Table 3), with a mean increase in methylation of 11.97% (methylation changes ranged from 3.5 to 29.6%), and a hypomethylation effect in low methylated genes, with a mean decrease in methylation of 58.54% (methylation changes ranged from 45.3 to 105.4%).

Using DNA methylation data from umbilical cord tissue and vastus lateralis biopsies we showed that *ACTBL2*, *BDNF_1, IL8*, and *MYH4* have similar methylation levels in both tissues (Supplementary Figures 1A,B; *p* > 0.05), and that *MYL6, OBSCN_4, BDNF_1, and PAX3* (*p* < 0.05) can be modified by resistance training in untrained male subjects (Supplementary Figure 1C).

**FIGURE 1:**
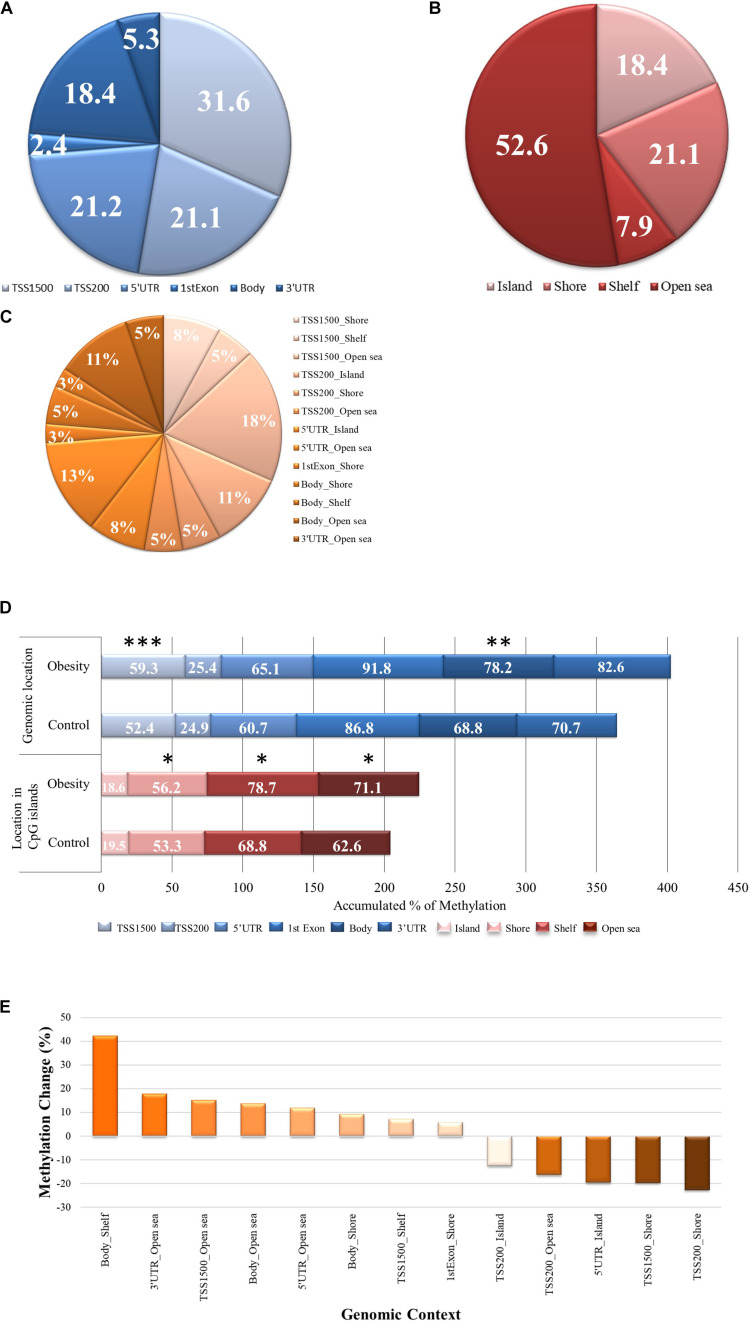
The CpGs methylation levels according to gene localization, localization in the CpG island and genomic context. **(A)** A pie chart showing the proportions of individual CpG sites according gene localization **(B)** A pie chart showing the proportions of individual CpG sites according localitzation in CpG island **(C)** A pie chart showing the proportions of individual CpG sites according genomic context **(D)** DNA methylation representation for individual CpG sites for all the studied gene localizations (TSS1500, TSS200, 5’UTR, 1stExon, Body, and 3’UTR) and localizations in CpG islands (island, shore, shelf and open sea) in gestational obesity and control group, using horizontal stacked graph bars. **(E)** A bar chart depicting the change in methylation percentage in gestational obesity in relation to the control group and according to genomic context. Each CpG site on the array is annotated to a genomic location (TSS1500, TSS200, 5′UTR, 1stExon, Body, and 3′UTR) and a location in the CpG Island (island, shore, shelf, and open sea) region. These were combined for each probe to form a unique genomic context annotation. *p < 0.0001; **p = 0.003, and ***p = 0.001. TSS, transcription start site; UTR, untranslated region.

From the analyzed individual CpG sites according to genomic location, 76% are located close to a transcription start site (TSS; Figure 1A). When examining the global methylation level according to individual CpG sites genomic localization between groups, we found that only CpGs within TSS1500 and gene body were highly methylated in the gestational obesity group compared to the control group (*p* = 0.001 and 0.003, respectively; Figure 1D). According to the location in CpG islands, 18.4% are in CpG islands, 21.2% in shores, 7.9% in shelves and 52.6 % in open sea (Figure 1B). When examining the global methylation level according to individual CpG sites within CpG islands, we found that CpGs within shores, shelves and open sea were highly methylated in the gestational obesity group compared to the control group (all *p*< 0.0001; Figure 1D). Finally, considering the genomic context (Figure 1C), hypermethylation can be seen mostly in locations distant to promoter regions and with lower CpG density while hypomethylation can be seen in locations closer to transcription sites and with a higher CpG density (Figure 1E).

### Gestational Obesity and Co-methylation Interactions

**FIGURE 2:**
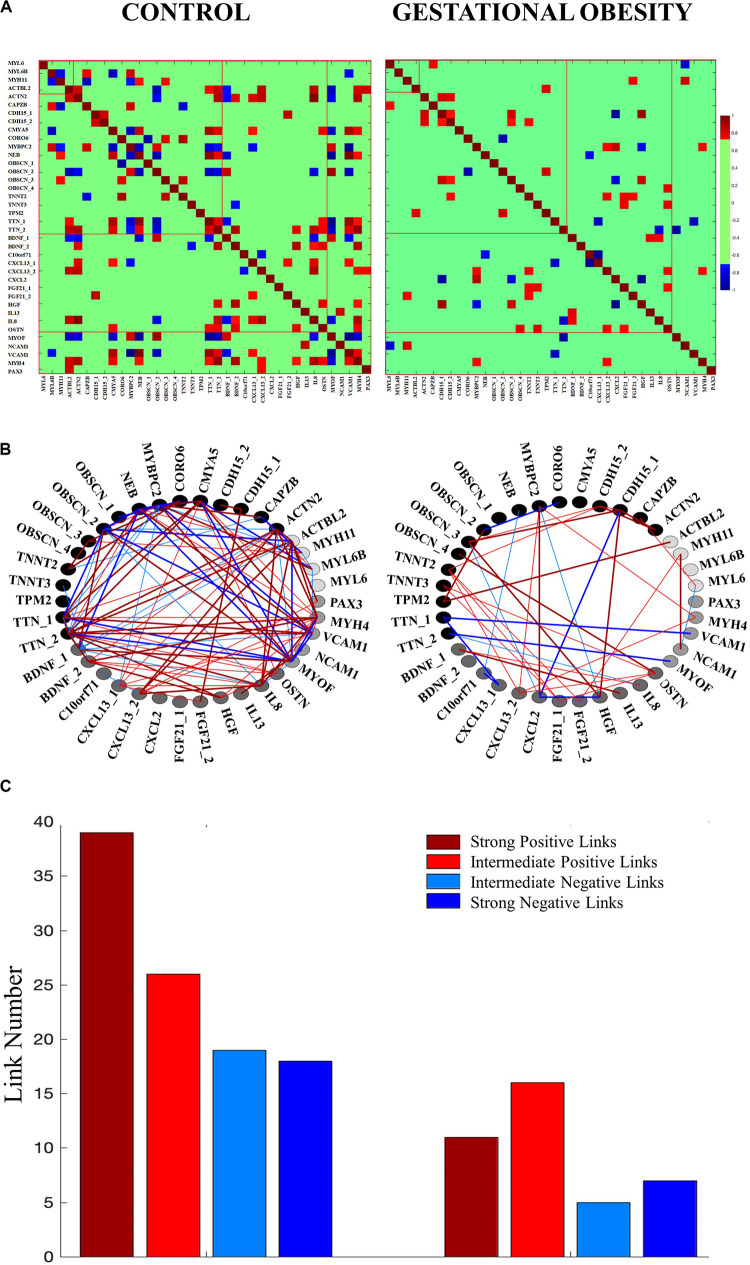
Co-methylation network interactions changes among the studied genes according to control and gestational obesity groups. **(A)** Gene co-methylation matrix. The matrices show the Pearson correlation coefficient of the methylation levels between every pair of genes. Non-significant correlations are represented in green. **(B)** Gene co-methylation networks. Each node represents a specific CpG for each studied gene category [contractibility (black), structure (dark gray), myokines (gray) and myogenesis (light gray)]. Links between two nodes represent the coupling strength (i.e., the Pearson coefficient) between two genes. The links are divided into four types: strong positive links (Pearson coefficients > 0.8; thick dark red lines), intermediate positive links (0.7 < Pearson coefficients < 0.8; thin red lines), intermediate negative links (−0.7 > Pearson coefficients >−0.8; thin blue lines), and strong negative links (Pearson coefficients <-0.8; thick dark blue lines). Note that the network only includes the significant correlations found in the co-methylation matrix. **(C)** Total number of links. The height of the bar corresponds to the number of significant correlations in the co-methylation matrix.

Our co-methylation networks showed that gestational obesity reduces the total number of interactions compared to the control group (Figure 2C). As for the different type of interactions, gestational obesity diminishes the number of all links types [SPL, by 47.5%, IPL, by 38%, INL, by 74%, and SNL, by 61% (Figure 2C)]. In the control group, positive associations were found among low methylated CpGs and among highly methylated CpGs, while negative associations were found between highly methylated CpGs and low methylated CpG. It is worth to note that gestation obesity changes not only the strength buy also the trend of the associations observed in structure and myokine gene categories (Figure 2).

**FIGURE 3:**
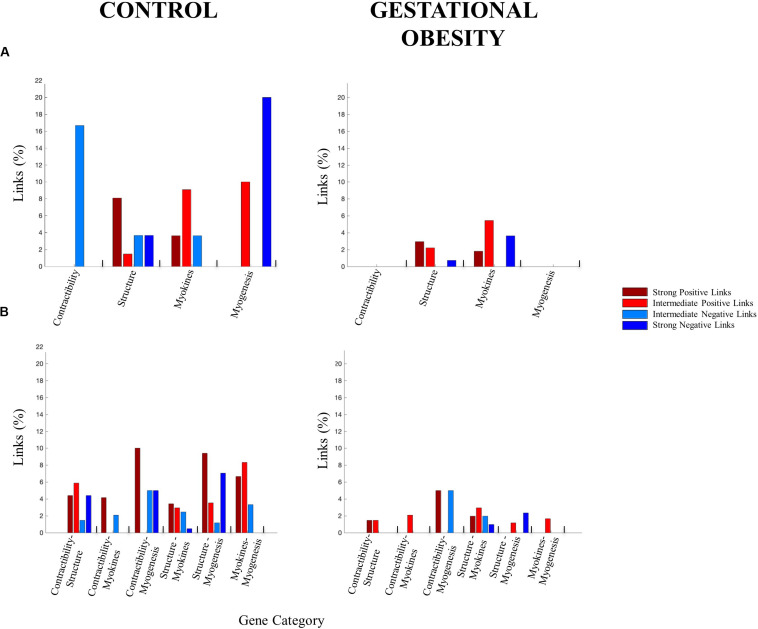
Percentage of links for **(A)** intra- gene categories and **(B)** inter-gene categories. The height of the bar corresponds to the percentage of significant correlations for each link type.

Interestingly, gestational obesity also changes both, the percentage of intra-gene category interactions (3.5% in average) and inter-gene category interactions (2.9% in average). Focusing on intra-gene category interactions (Figure 3A), gestational obesity causes a loss all types of links in contractibility and myogenesis categories. In the structure category, IPL are 100% higher in obesity compared to the control group while the SPL are reduced by 5%, the INL are lost, and the SNL are reduced by 3%. Finally, in the gestation obesity group the myokines category gain SNL while the INL are lost and a reduction of 2% in SPL and 4% IPL can be seen. Regarding the inter-gene category interactions (Figure 3B), the control group presents a higher number of all types of links in all gene categories. Gestational obesity in general promotes a diminution of the percentage of links between all gene categories and, furthermore, an absence of some type of links in the studied categories [Contractibility vs. structure (SPL decrease by 2.94%, IPL by 4.41%, INL by 1.47% and SPL by 4.41%), contractibility vs. myokines (SPL decrease by 4.17%, IPL increase by 2.08% and INL decrease by 2.08%), contractibility vs. myogenesis (SPL decrease by 5%, INL remain equal and SPL decrease by 5%), structure vs. myokines (SPL decrease by 1.47%, IPL remain equal, INL decrease by 0.49% and SPL increase by 0.49%), structure vs. myogenesis (SPI decrease by 9.41%, IPI by 2.35%, INI by 1.18% and SPI by 4.71%) and myokines vs. myogenesis (SPL decrease by 6.67%, IPL by 6.67% and INL by 3.33%)]. Note that, all type of links are maintained between structural and myokines CpG.

### DNA Methylation Levels Correlates With Anthropometric and Metabolic Variables in the Offspring

To test whether DNA methylation levels of skeletal muscle-specific genes in umbilical cord tissue may be associated with infant’s anthropometric and metabolic parameters at birth and at 6 years of age, Spearman’s correlation analysis between variables were performed (Supplementary Table 5).

At birth higher methylation levels of ACTBL2 (*r* = −0.518; *p* = 0.040) and IL8 (*r* = −0.538; *p* = 0.031) were associated with lower birth weight whereas a negative association between TPM2 methylation levels and ponderal index (*r* = −0.556; *p* = 0.025) was observed. However, the associations between methylation levels and birth anthropometry did not remain significant when correcting for multiple testing.

At 6 years of age, height SDS was positively associated with MYL6B (*r* = 0.627; *p* = 0.029), and negatively with TNNT3 (*r* = −0.616; *p* = 0.032), BMI SDS was positively associated with TTN_1, CXCL13_1 and, PAX3 (all from *r* = 0.650 to *r* = 0.580 and *p* = 0.022 to *p* = 0.047) and negatively associated with OBSCN_2, BDNF_2, and MYOF (all from *r* = −0.665 to *r* = −608 and *p* = 0.018 to *p* = 0.035). Fat mass percentage was associated positively with CXCL2 (*r* = 0.721, *p* = 0.018) and negatively with TNNT3 (*r* = −0.648; *p* = 0.042), and IL13 (*r* = −0.648; *p* = 0.042).

**FIGURE 4:**
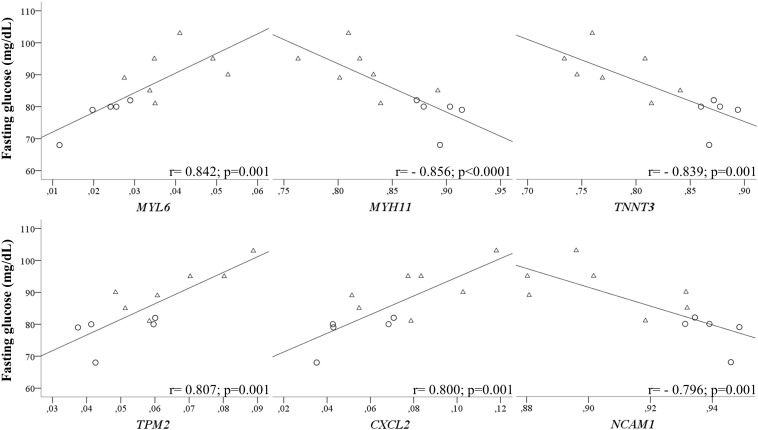
DNA methylation levels in umbilical cord tissue and fasting glucose levels at 6 years of age. Correlation graphs of DNA methylation levels of skeletal muscle-specific genes (*MYL6, MYH11, TNNT3, TPM2, CXCL2*, and *NCAM1*) in umbilical cord tissue with fasting glucose levels at 6 years of age. Triangles and circles depict subjects from control and gestational obesity group, respectively. *R-* and *p*-values are shown from Spearman correlation analysis.

Finally, fasting glucose at 6 years of age was positively associated with MYL6, MYL6B, CAPZB, MYBPC2, OBSCN_2, TPM2, BDNF_2, and CXCL2 (all from *r* = 0.842; to *r* = 0.684 and *p* = 0.001 to *p* = 0.014) and negatively associated with MYH11, CDH15_1, CDH15_2, CORO6, NEB, OBSCN_4, TNNT2, TNNT3, TTN_2, C10orf71, CXCL13_2, FGF21_1, IL13, OSTN, NCAM1, VCAM1, and MYH4 (all from *r* = −0.856; to *r* = −0.589 and *p* < 0.0001 to *p* = 0.043). Interestingly, the associations between fasting glucose and MYL6 (Contractibility; TSS200_Open sea), MYH11 (Contractibility; TSS200_Open sea), TNNT3 (Structure; Body_Shore), TPM2 (Structure; TSS200_Island), CXCL2 (Myokine; TSS200_Island), NCAM1 (Myogenesis; 5’UTR_Open sea) were still relevant after correcting for multiple testing (Figure 4 and Supplementary Table 5).

## Discussion

The percentage of methylation of at least 38 individual CpG sites within skeletal muscle-specific genes in umbilical cord tissue is modified by gestational obesity. Highly methylated genes in control samples showed hypermethylation in the gestational obesity group, while low methylated genes in control samples showed hypomethylation in the gestational obesity group. Interestingly, 76% of the identified individual CpG sites are close to a transcription starting site. On top of that, CpGs in regions close to transcription sites and with high CpG density are hypomethylated while regions distant to transcriptions sites and with low CpG density are hypermethylated in the gestational obesity group. In our networks, gestational obesity changed the intra- and inter-gene category interactions, reducing the number of all types of links in almost all the studied comparisons. We show for the first time that gestational obesity not only changes methylation levels but also changes the co-methylation pattern among genes coding for skeletal muscle in umbilical cord tissue. Finally, in our longitudinal analysis, DNA methylation levels of most of the skeletal muscle-specific genes were related to infant’s fasting glucose levels at age 6.

Sheep studies showed that gestational obesity led to increased fetal skeletal muscle mass with an impaired muscle quality (Tong et al., 2009). In line with that, maternal malnutrition of pregnant rats can affect the contractile properties (Bayol et al., 2005, 2007) the number and composition of muscle fibers (Sharples et al., 2016a) and muscle metabolism (Shelley et al., 2009; Simar et al., 2012) in the offspring. Our results suggest that gestational obesity can alter the methylation pattern of skeletal muscle-specific genes in umbilical cord tissue. Skeletal muscle can store and produce glucose, contributing to blood glucose homeostasis (Shieh et al., 2004). The endogenous glucose production rate and the susceptibility to hypoglycemia improve with age (Powell et al., 1981; Tsalikian et al., 1984; Collins et al., 1990) and this improvement has been related to the increase observed in muscle mass (Gallagher et al., 1999). At the same time, methylation levels of MYL6, MYH11, TNNT3, TPM2, CXCL2, and NCAM1 in umbilical cord tissue are related to infant’s fasting glucose levels measured in serum at 6 years of age. Fasting glucose is a glycemic measure with a “J-shaped” relationship with major cardiovascular events (Sarwar et al., 2010; Seshasai et al., 2011; Di Angelantonio et al., 2014; Zaccardi et al., 2015). While elevated glucose levels are a sign of reduced insulin secretion or action and also a proxy of type 2 diabetes (Tirosh et al., 2005; Nichols et al., 2008) and cardiovascular disease (Sarwar et al., 2010) lower levels of fasting glucose are associated with all-cause mortality (Bragg et al., 2016). Wändell suggested that low plasma glucose levels and BMI might serve as a marker of low fat-free mass, which in turn may imply a lower glucose reserve capacity and a higher susceptibility to develop different diseases (Wandell and Theobald, 2007). Interestingly, longitudinal cohort studying trajectories of fasting glucose have shown that this marker increases modestly over time in non-diabetic subjects (Andres and Tobin, 1975) suggesting that variation in fasting glucose is largely unaffected by age-related changes and may be established early in life (Barker et al., 2011). According to previous authors, skeletal muscle seems to retain and pass information from the environmental to daughter cells, through epigenetic processes (Sharples et al., 2016b). More interestingly, Davegårdh et al. (2017) cultured muscle stem cells from obese and non-obese subjects under identical conditions and observed a three-fold increase in the number of DNA methylation changes in the obese subjects. We herein suggest that the long-lasting effects of maternal obesity, at least on fasting glucose levels, could be mediated by an inappropriate epigenetic programming of skeletal muscle *in utero*. Further studies in larger cohort samples are needed to ascertain the use of these genes as early biomarkers of fasting glucose levels early in life.

In general, gene participation in a common pathway or functional similarity leads to gene co-methylation (Akulenko and Helms, 2013). Transcription factors (TFs) are proteins that bind to specific DNA sequences to regulate development and cell differentiation (Lee and Young, 2013). Several studies indicate that complex programs of cell differentiation might be regulated by a very small number of proteins (Weintraub et al., 1973; Holtzer et al., 1975a, b). In skeletal muscle, more than 170 TF have been identified. Among them, MyoD (the major TF that regulates muscle differentiation) can induce skeletal muscle differentiation in cells from many different lineages, including those from umbilical cord tissues (Davis et al., 1987; Weintraub et al., 1989; Gang et al., 2004). TEAD (1-4) (Jacquemin et al., 1996; Stewart et al., 1996) is also a TF which plays important roles in skeletal muscle differentiation, physiology, structure and contraction (Stewart et al., 1994; Yoshida, 2008). Our results show, that more than three quarters of the CpGs differently methylated in our study are close to a transcription starting site and are highly methylated in gestational obesity, compared to controls. Moreover, differently methylated CpGs were overrepresented at open seas and shelves and underrepresented at shores and islands. This is in accordance with studies aiming to describe disease-associated methylation patterns (Wang et al., 2012; Grundberg et al., 2013; Ollikainen et al., 2015). Overall, in our gestational obesity group, CpGs in regions close to transcription sites and with high CpG density are hypomethylated while regions distant to transcriptions sites and with low CpG density are hypermethylated. Our results could be in line with previous studies that demonstrated that obesity can alter epigenetic and transcriptomic regulation during differentiation in skeletal muscle (Tong et al., 2009). More studies are needed to clarify if indeed there exists a common regulator (e.g., TF) of skeletal muscle-specific genes than can be altered by gestational obesity.

Gestational obesity could perturb the normal skeletal muscle development physiology by the following mechanisms: (a) a decrease of the total number of interactions between CpGs, including the interactions intra- and inter- gene categories, and (b) changes in the type of interaction between CpGs (i.e., positive or negative), which can point toward to collaborative or antagonistic relations of these genes in skeletal muscle physiology. Our results suggest that gestational obesity would lead to a loss of complexity, at least for methylation of skeletal muscle-specific genes, revealing a complete reorganization of this methylation process in response to gestational obesity. Noteworthy, the only genes that maintain a similar number of interactions are those within genes related to structure and myokines, pointing to the fact that these genes could be the less affected functions by gestational obesity. As mentioned above, most skeletal muscle genes could be under the same methylation regulatory pathway, since methylation changes caused by gestational obesity seem to follow specifics patterns in our co-methylation networks interactions. The interactions defined in our network could have not only significant clinical implications in the offspring, but they could also be of relevance for the new emergent field of Network Physiology (Bashan et al., 2012; Bartsch et al., 2015; Lin et al., 2016). Understanding this differential methylation organization between normal and gestational obesity pregnancies could bring novel aspects of basic physiological regulation to skeletal muscle formation.

Most of the evidence to date that maternal excessive gestational weight gain affects skeletal muscle development and health into adult life comes from animal studies. There is a general lack of human data because methods to assess skeletal muscle are invasive and often require whole muscle dissection, which is not applicable to humans for obvious ethical reasons (Bayol et al., 2014). It is not known whether umbilical cord reflects the levels of methylation in skeletal muscle, but our results may suggest that umbilical cord tissue, an easily available tissue that can be sampled non-invasively, could be a potential marker of skeletal muscle features and fasting glucose levels. Using data from skeletal muscle biopsies in male subjects, we have shown that certain genes (*ACTBL2*, *BDNF_1, IL8, MYH4*) presents similar methylation levels in umbilical cord tissue compared with vastus lateralis biopsies and that methylation in *MYL6, OBSCN_4, BDNF_1, and PAX3* can be modified by both, obesity and resistance training. Previous studies have demonstrated that umbilical cord-derived mesenchymal stem cells could differentiate into a skeletal myogenic phenotype (Gang et al., 2004). Moreover, Murphy et al. (2012) reported that, at least for imprinted genes, methylation levels were comparable across multiple tissue types in humans (including umbilical cord and muscle) and emphasizes the potential utility of DNA methylation marks as early exposure assessment tools. However, more studies with longitudinal data are necessary to ascertain if the observed changes in umbilical cord tissue are related to skeletal muscle physiology and global health in childhood and adulthood. And if those genes affected by gestational obesity can also be affected by both, resistance and endurance training.

We acknowledge a number of study limitations. Longitudinal studies with a higher number of participants are needed to confirm the role of gestational obesity in the epigenetic status of muscle-specific genes in umbilical cord tissue. We could not perform skeletal muscle biopsies in the children included in our study due to ethical reasons, but it would be interesting to compare the methylation profile in normal cord with skeletal muscle in animal studies. The possible effect of gestational obesity on skeletal muscle tissue and its implications for children development and adult metabolic diseases would be interesting to explore in the future. We selectively studied genes associated with DNA methylation changes but genes important in other lineages or tissues not necessarily regulated by DNA methylation could be also affected by gestational obesity and should be further studied. Finally, other factors, such as diet or physical activity, which were not available for the current study, should be considered in future studies as possible confounders for co-methylation interactions studies.

In conclusion, our study showed a complex interaction between gestational obesity and the epigenetic status of muscle-specific genes in umbilical cord tissue. Additionally, gestational obesity may alter the functional co-methylation connectivity of CpG within skeletal muscle-specific genes interactions, our results revealing an extensive reorganization of methylation in response to maternal overweight. Finally, changes in methylation levels of skeletal muscle specific genes may have persistent effects on the offspring of mothers with gestational obesity.

## Data Availability Statement

The DNA methylation data has been deposited into GEO (Accession: GSE153564), https://www.ncbi.nlm.nih.gov/geo/query/acc.cgi?acc=GSE153564.

## Ethics Statement

The studies involving human participants were reviewed and approved by the Ethics Committee of Clinical Research (CEIC) of Dr. Josep Trueta Hospital. The patients/participants provided their written informed consent to participate in this study.

## Author Contributions

AP-P, FV, SG-R, MP-P, SX-T, BM-P, RF-L, JB, and AL-B conceived the experiments and analyzed the data. AP-P, FV, SX-T, GC-B, BM-P, and JB carried out the experiments. SG-R, JB, and AL-B critically contributed to the manuscript revision. AL-B supervised the work. All authors were involved in writing the manuscript and approved the submitted and published versions.

## Conflict of Interest

The authors declare that the research was conducted in the absence of any commercial or financial relationships that could be construed as a potential conflict of interest.
