# Supplementary figures and images for: DNA Methylation Reorganization of Skeletal Muscle-Specific Genes in Response to Gestational Obesity
Source: Front Physiol. 2020 Jul 31;11:938. doi: 10.3389/fphys.2020.00938 (PMC7412435; doi:10.3389/fphys.2020.00938)

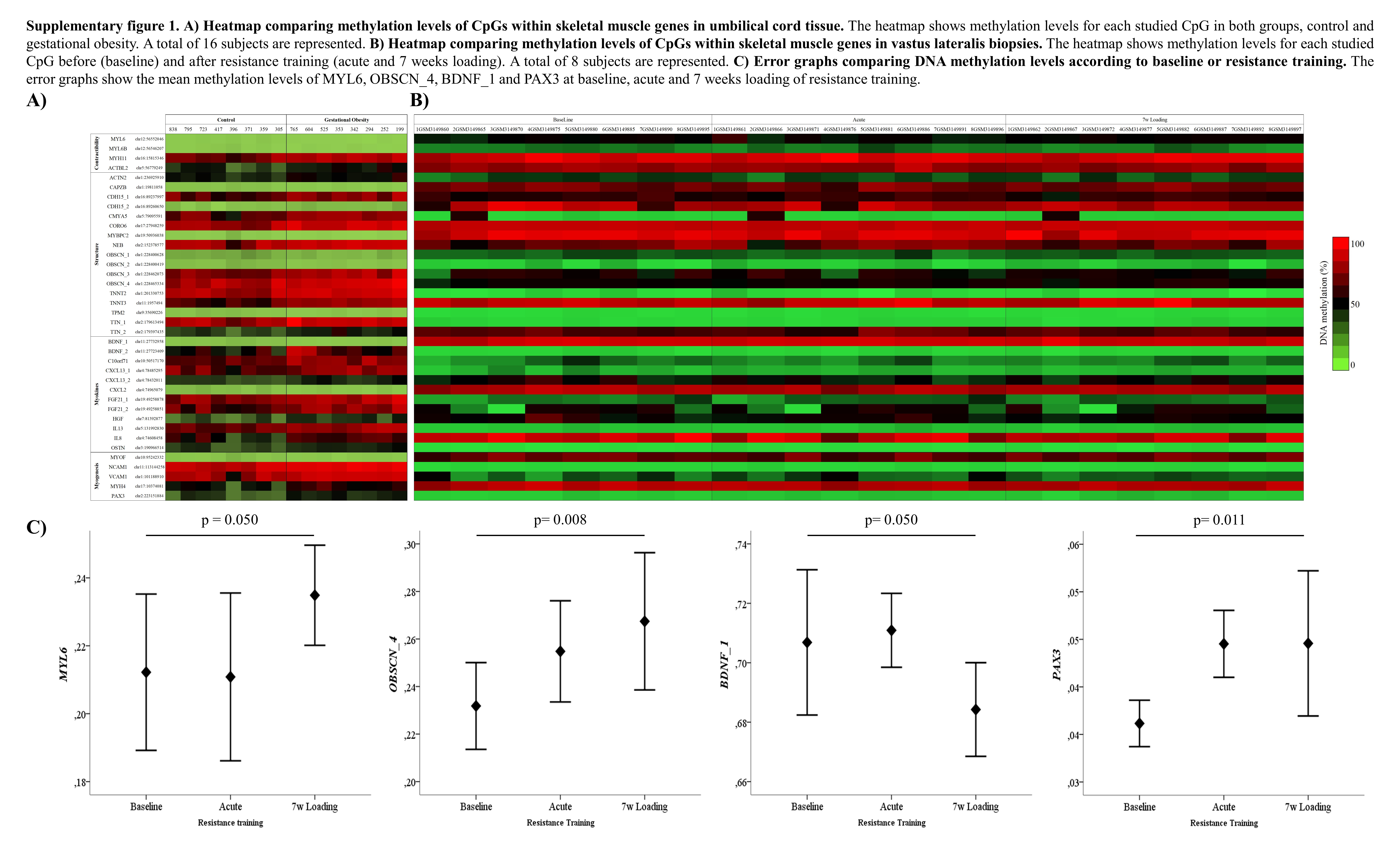

Supplement: Supplementary file 1 [file Image_1.jpg]
