# Supplementary material for: DNA Methylation Reorganization of Skeletal Muscle-Specific Genes in Response to Gestational Obesity
Source: Front Physiol. 2020 Jul 31;11:938. doi: 10.3389/fphys.2020.00938 (PMC7412435; doi:10.3389/fphys.2020.00938)
Supplement: Supplementary file 2 [file Table_1.DOCX]

Supplementary Material

# Supplementary Tables

**Supplementary Table 1:** Clinical assessments in the studied subjects (n=16).

|  | **Control** | **Gestational obesity** | **p-value** |
| --- | --- | --- | --- |
| N | 8 | 8 | - |
| **Pregnant Mother** | | | |
| Age (years) | 32.0 ± 4.4 | 30.6 ± 3.1 | 0.505 |
| BMI before pregnancy (kg/m^2^) | 21.4 ± 2.0 | 22.3 ± 1.7 | 0.505 |
| **Weight increase during pregnancy (kg)** | **14.3 ± 0.9** | **19.2 ± 2.6** | **<0.0001** |
| Gestational Age (weeks) | 40.1 ± 1.2 | 40.2 ± 0.4 | 1.000 |
| **Newborn** | | | |
| Ponderal Index (g/cm^3^) | 2.72 ± 0.27 | 2.71 ± 0.14 | 0.529 |
| Weight Birth SDS | 0.16 ± 0.72 | -0.04 ± 0.72 | 0.442 |
| Height Birth SDS | -0.02 ± 0.80 | -0.27 ± 0.46 | 0.382 |
| Blood cord Insulin (mlU/L) | 5.69 ± 4.55 | 5.15 ± 4.6 | 0.874 |
| **At 6 years of age (n=12, 6 per group)** | | | |
| Weight SDS | 0.11 ± 0.88 | 0.10 ± 0.82 | 0.685 |
| Height SDS | 0.91 ± 0.74 | -0.02 ± 0.49 | 0.167 |
| BMI SDS | -0.39 ± 0.77 | 0.10 ± 0.57 | 0.169 |
| Fat Mass (%) | 24.8 ± 6.4 | 16.9 ± 5.9 | 0.114 |
| **Fasting glucose (mg/dL)** | **91.1 ± 7.2** | **77.8 ± 5.5** | **0.007** |

BMI: body mass index;SDS: standard deviation score.

Data are expressed as mean ± SD. P-values are form Mann-Whitney U test.

**Supplementary Table 2:** CpGs associated with skeletal muscle according to previous literature and presented grouped by categories.

| **CSC RefGene Name** | **CpG Position (GRCh37)** | **FDR** | **OR** |
| --- | --- | --- | --- |
| **Contractibility** | | | |
| ACTBL2 | chr5:56779120 | 0,00123583 | 1,3722163 |
|  | **chr5:56779249** | **0,00154906** | **1,83509131** |
| MYL6 | **chr12:56552046** | **0,00018013** | **0,58796478** |
| MYL6B | **chr12:56546207** | **0,00148057** | **0,65664278** |
| NDE1;MYH11 | **chr16:15815346** | **3,17E-07** | **1,696509** |
|  | chr16:15819906 | 1,65E-05 | 1,34041906 |
| **Structure** | | | |
| ACTN2 | **chr1:236925910** | **0,00111844** | **1,51659276** |
| BAG3 | chr10:121432113 | 0,00103718 | 1,25757773 |
| CAPZB | chr1:19813436 | 0,00015104 | 1,46442881 |
|  | chr1:19811322 | 0,00041483 | 0,75674349 |
|  | chr1:19669284 | 0,00021119 | 1,40111185 |
|  | **chr1:19811058** | **7,34E-05** | **0,6563406** |
|  | chr1:19670446 | 0,00121407 | 1,4016532 |
| CDH15 | **chr16:89237997** | **0,00126456** | **1,78269197** |
|  | chr16:89237370 | 0,00032305 | 0,84314903 |
|  | **chr16:89260650** | **0,00040513** | **1,52984018** |
| CMYA5 | chr5:78985588 | 0,00073442 | 0,75935934 |
|  | **chr5:79095591** | **0,0003397** | **1,65354916** |
| CORO6 | **chr17:27948259** | **2,32E-05** | **1,6828113** |
| DES | chr2:220289908 | 1,69E-05 | 1,466342 |
| LRCH4;ZASP | chr7:100173244 | 0,00028068 | 1,38824923 |
| MYBPC2 | **chr19:50936038** | **0,00036253** | **0,52472547** |
|  | chr19:50936058 | 0,00029615 | 0,68950712 |
| MYOM2 | chr8:2067030 | 0,00035035 | 1,31567329 |
|  | chr8:2021126 | 0,00161652 | 1,36607098 |
|  | chr8:2029388 | 0,00121057 | 1,13263072 |
| MYOM3 | chr1:24423818 | 1,90E-05 | 1,47640774 |
|  | chr1:24404258 | 0,00016495 | 1,48353263 |
| NEB | **chr2:152378577** | **0,00118948** | **1,53943471** |
| OBSCN | chr1:228503273 | 0,00014719 | 1,37364674 |
|  | **chr1:228400628** | **7,10E-05** | **0,67015352** |
|  | chr1:228546589 | 0,00151154 | 1,36994416 |
|  | chr1:228495060 | 0,00020839 | 1,26641266 |
|  | chr1:228473704 | 0,00089884 | 1,11254576 |
|  | **chr1:228400419** | **0,000178** | **0,6527465** |
|  | chr1:228524822 | 0,00015957 | 1,37754626 |
|  | **chr1:228462073** | **0,00030525** | **1,56240657** |
|  | chr1:228506283 | 0,0003518 | 1,35315402 |
|  | chr1:228548163 | 1,55E-05 | 1,2923071 |
|  | **chr1:228465334** | **0,00035839** | **1,52734244** |
|  | chr1:228473781 | 0,00131527 | 1,34545966 |
|  | chr1:228492332 | 0,00081983 | 1,4014505 |
|  | chr1:228548266 | 0,00145873 | 1,2563218 |
|  | chr1:228555184 | 0,0002551 | 1,49953067 |
|  | chr1:228466523 | 5,72E-09 | 1,32679945 |
|  | chr1:228443868 | 0,00140863 | 1,38624274 |
|  | chr1:228504761 | 0,00022136 | 1,34351476 |
|  | chr1:228394900 | 0,00093272 | 1,30714182 |
| OBSL1 | chr2:220432822 | 0,00092675 | 1,37855026 |
|  | chr2:220415535 | 0,00010403 | 1,30713878 |
| TNNT2 | chr1:201334006 | 0,00135834 | 1,38049785 |
|  | chr1:201344123 | 0,00045728 | 1,43861808 |
|  | chr1:201348231 | 0,0001805 | 1,2980596 |
|  | chr1:201336121 | 0,0012742 | 1,37578798 |
|  | chr1:201333484 | 0,00034873 | 1,46232343 |
|  | chr1:201347529 | 0,00085834 | 1,46229071 |
|  | chr1:201347550 | 0,00094354 | 1,32790899 |
|  | **chr1:201330753** | **0,00044751** | **1,52073798** |
|  | chr1:201337540 | 0,00092746 | 1,28250133 |
| TNNT3 | chr11:1959051 | 0,00064717 | 1,37474043 |
|  | **chr11:1957494** | **2,81E-10** | **1,78752197** |
|  | chr11:1950354 | 0,00110855 | 1,37577835 |
|  | chr11:1958316 | 7,21E-06 | 1,26483399 |
| TPM1 | chr15:63334506 | 0,00063225 | 0,81400509 |
| TPM2 | **chr9:35690226** | **0,00131845** | **0,67488812** |
| TPM3 | chr1:154164994 | 0,0010467 | 1,23092327 |
| TTN | **chr2:179613494** | **0,00111785** | **1,8936726** |
|  | **chr2:179397435** | **0,00032375** | **1,63494064** |
|  | chr2:179500956 | 0,00080455 | 1,41353576 |
|  | chr2:179516258 | 4,18E-06 | 1,49446159 |
| ZDHHC24;ACTN3 | chr11:66312752 | 0,00068121 | 1,36216405 |
| **Myokine** | | | |
| BDNF | chr11:27722549 | 0,00017224 | 0,77779415 |
|  | chr11:27723237 | 2,78E-05 | 0,74287806 |
|  | **chr11:27732958** | **0,00049847** | **2,01924937** |
|  | **chr11:27723409** | **2,27E-05** | **0,6690423** |
|  | chr11:27679632 | 0,00088401 | 1,39849201 |
| C10orf71 | chr10:50515644 | 0,00063751 | 1,26152202 |
|  | **chr10:50517170** | **2,56E-06** | **1,51221414** |
| C1QTNF5;MFRP | chr11:119215337 | 9,47E-05 | 1,33884637 |
|  | chr11:119212949 | 0,00027879 | 1,27250062 |
| CXCL1 | chr4:74735924 | 0,00043004 | 1,33503648 |
| CXCL13 | **chr4:78485295** | **0,00023125** | **1,58334104** |
|  | **chr4:78432011** | **0,00132509** | **1,52182968** |
| CXCL14 | chr5:134915080 | 0,00152118 | 0,79404948 |
| CXCL2 | **chr4:74965079** | **0,00019656** | **0,60672371** |
| CXCL5 | chr4:74865528 | 2,93E-06 | 1,46348508 |
| FGF21;FUT1 | **chr19:49258878** | **0,00029995** | **1,51222605** |
|  | **chr19:49258851** | **0,00026365** | **1,60562461** |
| FNDC5 | chr1:33334878 | 0,00050892 | 1,4134196 |
| HGF | **chr7:81392877** | **0,00059166** | **1,90729249** |
| IL10 | chr1:206946595 | 0,00021791 | 1,48658314 |
| IL15 | chr4:142623193 | 0,0008331 | 1,33084655 |
| LIF | chr22:30639730 | 0,00016178 | 1,25501651 |
| OSTN | **chr3:190966514** | **0,00020787** | **1,67669737** |
| TH2LCRR;IL13 | **chr5:131992830** | **3,77E-06** | **1,51528722** |
| ZMYND15;CXCL16;ZMYND15 | chr17:4644276 | 0,00137614 | 1,32751405 |
| BDNF | chr11:27743580 | 9,17E-05 | 1,26672376 |
| IL8 | **chr4:74608458** | **0,00085323** | **2,03045513** |
| IL15 | chr4:142635753 | 3,98E-05 | 1,49377248 |
| **Myogenesis** | | | |
| LCAT | chr16:67976842 | 0,00058651 | 1,38968686 |
| MHRT;MYH7 | chr14:23884196 | 0,00153799 | 1,29081951 |
| MYH3 | chr17:10559323 | 0,00117448 | 1,44266207 |
| MYH4;MYHAS | **chr17:10374081** | **0,00082691** | **1,96412867** |
| MYH7 | chr14:23884544 | 8,02E-06 | 1,41832598 |
|  | chr14:23903787 | 0,00022897 | 1,43567948 |
| MYOF | **chr10:95242332** | **0,00108041** | **0,64963347** |
|  | chr10:95242111 | 0,00144202 | 0,80292948 |
|  | chr10:95242154 | 0,00136036 | 0,76774998 |
| NCAM1 | **chr11:113144258** | **1,38E-05** | **1,59104272** |
| NFIX | chr19:13144918 | 0,00047015 | 1,44298349 |
|  | chr19:13149516 | 0,00145558 | 1,34614502 |
| PAX3 | **chr2:223151884** | **0,00012739** | **2,06466126** |
|  | chr2:223159838 | 0,00135859 | 0,85315993 |
| PAX7 | chr1:18959625 | 0,00040109 | 0,75468435 |
| VCAM1 | **chr1:101188910** | **0,00140245** | **1,65557005** |

**Supplementary Table 3:** Methylation levels of the studied CpGs according to control and gestational obesity groups.

|  |  | **Genomic**  **Context** | **Methylation levels** | | **Methylation change (%)** |  |
| --- | --- | --- | --- | --- | --- | --- |
|  |  |  | Control | Gestational Obesity |  | **p-value** |
| **Contractibility** | MYL6 | TSS200_Open sea | 3.83 ± 0.01 | 2.27 ± 0.01 | -68.4 | 0.001 |
|  | MYL6B | TSS200_Island | 4.04 ± 0.01 | 2.62 ± 0.01 | -54.0 | 0.010 |
|  | MYH11 | TSS200_Open sea | 82.5 ± 0.03 | 89 ± 0.01 | 7.3 | 0.002 |
|  | ACTBL2 | TSS1500_Open sea | 51.5 ± 0.14 | 66.4 ± 0.05 | 22.3 | 0.050 |
| **Structure** | ACTN2 | Body_Open sea | 60.9 ± 0.07 | 70.4 ± 0.04 | 13.5 | 0.01 |
|  | CAPZB | TSS1500_Shore | 6.19 ± 0.01 | 4.15 ± 0.01 | -49.3 | 0.002 |
|  | CDH15_1 | TSS200_Island | 79.1 ± 0.03 | 85.3 ± 0.03 | 7.2 | 0.007 |
|  | CDH15_2 | Body_Shore | 6.12 ± 0.02 | 9.51 ± 0.02 | 35.6 | 0.015 |
|  | CMYA5 | 3'UTR_Open sea | 78.6 ± 0.07 | 86.5 ± 0.01 | 9.1 | 0.001 |
|  | CORO6 | 1stExon_Shore | 86.7 ± 0.02 | 91.8 ± 0.01 | 5.4 | 0.002 |
|  | MYBPC2 | TSS200_Shore | 4.44 ± 0.02 | 2.16 ± 0.01 | -105.4 | 0.002 |
|  | NEB | Body_Open sea | 86.7 ± 0.04 | 91.4 ± 0.01 | 5.1 | 0.001 |
|  | OBSCN_1 | 5'UTR_Island | 15.7 ± 0.03 | 10.8 ± 0.02 | -45.3 | 0.007 |
|  | OBSCN_2 | 5'UTR_Island | 8.47 ± 0.01 | 5.74 ± 0.01 | -47.3 | 0.002 |
|  | OBSCN_3 | 5'UTR_Island | 83.4 ± 0.02 | 88.4 ± 0.02 | 5.6 | 0.007 |
|  | OBSCN_4 | TSS200_Shore | 88.2 ± 0.03 | 92.2 ± 0.01 | 4.3 | 0.021 |
|  | TNNT2 | TSS1500_Open sea | 87.9 ± 0.02 | 91.9 ± 0.01 | 4.3 | 0.005 |
|  | TNNT3 | Body_Shore | 78.3 ± 0.03 | 86.7 ± 0.01 | 9.7 | <0.0001 |
|  | TPM2 | TSS200_Island | 6.38 ± 0.01 | 4.35 ± 0.01 | -46.5 | 0.010 |
|  | TTN_1 | TSS1500_Open sea | 87.2 ± 0.03 | 91.7 ± 0.02 | 4.9 | 0.001 |
|  | TTN_2 | 5'UTR_Open sea | 47.5 ± 0.09 | 63.3 ± 0.07 | 24.9 | 0.001 |
| **Myokine** | BDNF_1 | 5'UTR_Open sea | 6.53 ± 0.01 | 4.4 ± 0.01 | -48.4 | 0.002 |
|  | BDNF_2 | TSS1500_Shore | 66.8 ± 0.11 | 80.3 ± 0.07 | 16.8 | 0.010 |
|  | C10orf71 | 5'UTR_Open sea | 80.1 ± 0.02 | 85.7 ± 0.03 | 6.5 | 0.002 |
|  | CXCL13_1 | 5'UTR_Open sea | 79.2 ± 0.05 | 85.6 ± 0.02 | 7.4 | 0.021 |
|  | CXCL13_2 | TSS1500_Open sea | 48.6 ± 0.05 | 59.9 ± 0.07 | 18.8 | 0.021 |
|  | CXCL2 | TSS200_Island | 8.2 ± 0.02 | 5.02 ± 0.01 | -63.1 | 0.003 |
|  | FGF21_1 | TSS1500_Shelf | 84.5 ± 0.03 | 89.1 ± 0.02 | 5.2 | 0.005 |
|  | FGF21_2 | TSS1500_Shelf | 79.1 ± 0.04 | 86 ± 0.03 | 8.0 | 0.021 |
|  | HGF | TSS1500_Open sea | 38.5 ± 0.11 | 54.4 ± 0.09 | 29.2 | 0.010 |
|  | IL13 | TSS1500_Shore | 81.5 ± 0.02 | 86.9 ± 0.02 | 6.2 | 0.001 |
|  | IL8 | 3'UTR_Open sea | 62.7 ± 0.15 | 78.7 ± 0.02 | 20.3 | 0.015 |
|  | OSTN | Body_Open sea | 47.7 ± 0.09 | 60.6 ± 0.04 | 21.1 | 0.010 |
| **Myogenesis** | MYOF | TSS1500_Open sea | 6.26 ± 0.02 | 3.97 ± 0.01 | -57.7 | 0.010 |
|  | NCAM1 | 5'UTR_Open sea | 90.6 ± 0.02 | 94 ± 0.01 | 3.5 | 0.001 |
|  | VCAM1 | Body_Open sea | 85.4 ± 0.07 | 91.4 ± 0.01 | 6.4 | 0.001 |
|  | MYH4 | TSS1500_Open sea | 59.7 ± 0.15 | 75.2 ± 0.03 | 20.6 | 0.001 |
|  | PAX3 | Body_Shelf | 43 ± 0.11 | 61.1 ± 0.05 | 29.6 | 0.005 |

TSS: Transcription Starting Site; UTR: untranslated region. Data represent the mean ± SD. P-values are form Mann-Whitney U test.

**Supplementary Table 4:** Known function and their defined role related to skeletal muscle for each of the studied gene. Data are presented according to gene categories.

|  | **UCSC RefGene Name** | **Full name** | **Gene Function** | **Relation SkM** | **References** |
| --- | --- | --- | --- | --- | --- |
| **Contractibility** | MYL6 | Myosin light chain 6 | Muscle filament sliding, muscle contraction, structural constituent of a muscle, skeletal muscle tissue development | Muscle filament sliding, muscle contraction, structural constituent of a muscle, skeletal muscle tissue development | Heissler & Sellers, 2014 |
|  | MYL6B | Myosin light chain 6B | Improves muscle contractibility by controlling muscle filament sliding and muscle contraction, has a structural constituent role, and is involved in skeletal muscle tissue development | Improves muscle contractibility by controlling muscle filament sliding and muscle contraction, has a structural constituent role, and is involved in skeletal muscle tissue development | Pillon, 2020 |
|  | MYH11 | Myosin heavy chain 11 | Actin filament binding and muscle contraction. Regulates neuron production, involved in chromosome segregation and establishment of chromosome localization. | Muscle contractibility and motor activity regulation; Structural constituent of a muscle, contractile function - converting chemical energy into mechanical, through the hydrolysis of ATP. | Munters, 2016 |
|  | ACTBL2 | Actin beta like 2 | Cellular motility, ATP binding, acts as a structural constituent of the contractile apparatus | Filament sliding and muscle contraction, a major constituent of the contractile apparatus of a muscle | Nieman & Pence, 2019 |
| **Structure** | ACTN2 | Actinin alpha 2 | Crosslink filamentous actin molecules - F-actin cross-linking function | Structural constituent - actin filament, Z disc of a muscle sarcomere, sarcomere organization, regulates skeletal muscles' membrane potential via Na-K pump, muscle filament sliding function, a role in muscle contraction | Gautel, 2016 |
|  | CAPZB | Capping actin protein of muscle Z-line subunit beta | Regulates the growth of actin filaments, cell morphology and cytoskeleton organization | Act as a structural component of a sarcomere, regulates cell morphology and cytoskeleton organization, controls the growth of the actin filaments | Mukherjee, 2016 |
|  | CDH15 | Cadherin 15 | Provides trigger for terminal muscle differentiation, controls myogenesis | Controls the process of morphogenesis in skeletal muscle, growth and improved function of skeletal muscle, regulates skeletal muscle differentiation | Kim, 2018 |
|  | CMYA5 | Cardiomyopathy associated 5; Myospryn | Negatively modulates skeletal muscle regeneration, assembles ryanodine receptor clusters in a striated muscle. | Act as a large scaffolding protein localized to the Z-disc region of striated muscle | Kielbasa, 2011 |
|  | CORO6 | Coronin 6 | Actin filament binding function | Cell migration and proliferation, actin filament binding and organization, and structural constituent function | Cardinali, 2016 |
|  | MYBPC2 | Myosin binding protein C2 | Plays structural role and modulates muscle contraction | Structural constituent of a muscle, filament binding function, responsible for muscle filament sliding, and has an important role in modulating muscle contraction | Lin,2018 |
|  | NEB | Nebulin | Binds and stabilizes F-actin, maintains a structural integrity of sarcomers and membranes of a myofibril | Structural constituent of a muscle cell, maintains structural integrity of a sarcomere, involved in filament sliding mechanism, provides muscle contraction and skeletal muscle development | Gautel, 2016 |
|  | OBSCN | Obscurin, cytoskeletal calmodulin and titin-interacting RhoGEF | Acts as a structural component of a striated muscle, has a role in myofibrinogenesis | Structural function, sarcomere organization function, and involved in myofibrinogenesis | Gautel, 2016 |
|  | TNNT | Troponin T3, fast skeletal type | Confers calcium-sensitivity to striated muscle actomyosin ATPase activity, calcium ion binding and tropomyosin binding function | Structural component, sarcomere organization and skeletal muscle structural function, filament sliding mechanism, and muscle contraction regulation | Stavroulakis & George, 2020 |
|  | TPM2 | Tropomyosin 2 | Binds to actin filaments in muscle and non-muscle cells, regulates muscle contraction | Structural constituent of a muscle, filament binding and organization, muscle filament sliding function, and muscle contraction regulation | Meng, 2019 |
|  | TTN | Titin | Provides connections in individual microfilaments, regulates sarcomere extensibility in a muscle, involved in chromosome condensation and segregation during mitosis. | Structural constituent of a muscle, filament and sarcomere organization, filament sliding function, muscle contraction regulation by controlling the sarcomere extensibility in a muscle | Gautel, 2016 |
| **Myokine** | BDNF | Brain derived neurotrophic factor | Support survival of existing neurons, encourage growth and differentiation of new neurons and synapses | Enhances fat oxidation in skeletal muscle, influences skeletal muscle fiber-type specification by elevating the glycolytic fiber number, provides muscle repair and muscle regeneration, myogenesis | Pedersen, 2013 |
|  | C10orf71 | Chromosome 10 open reading frame 71 | Plays an important role in cardiomyocyte hypertrophy via activation of the calcineurin/NFAT signaling pathway | Activates the calcineurin/NFAT signaling pathway and it is a cellular component of skeletal muscles' Z disc | Dierck, 2017 |
|  | CXCL13 | C-X-C motif chemokine ligand 13 | B lymphocyte chemoattractant; controls the migration of B lymphocytes | Cell signaling pathways in skeletal muscle, muscular angiogenesis, muscle regeneration modulated by muscle contraction-induced inflammation as an immune response | Griffin, 2010 |
|  | CXCL2 | C-X-C motif chemokine ligand 2 | A powerful neutrophil chemoattractant; encodes secreted proteins involved in immunoregulatory and inflammatory processes | Myoblast migration involved in muscle regeneration, signaling process in skeletal muscle cells, skeletal muscle angiogenesis, inflammatory and immune response in skeletal muscles | Pillon, 2020 |
|  | FGF21 | Fibroblast growth factor 21; fucosyltransferase 1 | This protein is a secreted endocrine factor that functions as a major metabolic regulator; stimulates glucose uptake in adipocytes. | Increases fatty acid oxidation, tricarboxylic acid cycle flux, and gluconeogenesis without increasing glucogenesis; involved in carbohydrate metabolic process into the skeletal muscle | Pedersen, 2013 |
|  | HGF | Hepatocyte growth factor | Regulates cell growth, cell motility, and morphogenesis in numerous cell and tissue types, | Regulates the metabolic flux of glucose in skeletal muscle, mediates signaling pathway in a muscle, induces muscle cell morphogenesis and muscle cell growth and proliferation | Leal, 2018 |
|  | IL13 | T helper type 2 locus control region associated RNA; Interleukin 13 | Produces long non-coding RNAs; Induces proliferation and immunoglobulin E (IgE) synthesis by human B cells. This cytokine down-regulates macrophage activity, thereby inhibits the production of pro-inflammatory cytokines and chemokines. | Cell signaling and immune response in muscle cells; Muscle regeneration modulated by the immune and inflammatory mechanisms in skeletal muscle | Leal, 2018 |
|  | IL8 | Interleukin 8; C-X-C motif chemokine ligand 8 | [Induces chemotaxis in neutrophils and other granulocytes, and stimulates phagocytosis](https://en.wikipedia.org/wiki/Chemotaxis) | Mediates signaling pathway and stimulates angiogenesis in skeletal muscle, modulates immune and inflammatory response as a part of muscle regeneration | Pedersen, 2013 |
|  | OSTN | Osteocrin | Regulates dendritic growth in the developing cerebral cortex in response to sensory experience | Regulates mitochondrial biogenesis in skeletal muscle | Subbotina,2015 |
| **Myogenesis** | MYOF | Myoferlin | Has a role in calcium mediated membrane fusion events - membrane regeneration and repair | Myoblast fusion and muscle fiber development, regulates muscle contraction | Doherty, 2005 |
|  | NCAM1 | Neural cell adhesion molecule 1 | Neuron-neuron adhesion,neurite fasciculation, outgrowth of neurites | Regulates cell to cell interactions, induces muscle differentiation and development | Chal, 2017 |
|  | VCAM1 | Vascular cell adhesion molecule 1 | Leukocyte-endothelial cell adhesion - mediates the adhesion of lymphocytes, monocytes, eosinophils and basophiles to vascular endothelium | Mediates signaling pathways, involved in myogenesis and muscle regeneration | Chal, 2017 |
|  | MYH4 | Myosin heavy chain 4; Myosin heavy chain gene cluster antisense RNA | Encodes a sarcomeric myosin; Produces long non-coding RNA that plays a role in muscle development | Plays a role in muscle development. Structural component of a muscle fiber, plays a role in sarcomere organization, provides muscle contraction and extensibility; Responsible for filament sliding mechanism and muscle contraction | Chal, 2017 |
|  | PAX3 | Paired box 3 | Regulate cell proliferation, migration and apoptosis - neural development and myogenesis | Involved in the process of myogenesis and muscle development, by regulating cell proliferation mechanism | Chal, 2017 |

**Supplementary table 5:** Spearman’s correlation between DNA methylation levels of skeletal muscle-specific genes in umbilical cord tissue and anthropometric and metabolic parameters at birth and at 6 years.

|  |  |  | **At birth variables (n=16)** | | | | **At 6 years of age variables (n=12)** | | | | |
| --- | --- | --- | --- | --- | --- | --- | --- | --- | --- | --- | --- |
|  |  |  | Ponderal Index (g/cm3) | Weight SDS Birth | Height SDS Birth | Cord Blood Insulin (mlU/L) | Weight SDS | Height SDS | BMI SDS | Fat mass (%) | Fasting glucose (mg/dL) |
| **Contractibility** | *MYL6* | r | -0.126 | 0.161 | 0.014 | 0.061 | 0.125 | 0.388 | -0.244 | 0.624 | ***0.842*** |
|  |  | p | 0.641 | 0.549 | 0.956 | 0.823 | 0.696 | 0.211 | 0.443 | 0.053 | ***0.001*** |
|  | *MYL6B* | r | -0.035 | 0.002 | -0.071 | 0.008 | 0.286 | **0.627** | -0.041 | 0.515 | **0.782** |
|  |  | p | 0.896 | 0.991 | 0.793 | 0.974 | 0.366 | **0.029** | 0.896 | 0.127 | **0.002** |
|  | *MYH11* | r | 0.329 | 0.167 | 0.079 | -0.360 | -0.167 | -0.518 | 0.251 | -0.296 | ***-0.856*** |
|  |  | p | 0.212 | 0.534 | 0.768 | 0.171 | 0.602 | 0.084 | 0.429 | 0.404 | ***<0.0001*** |
|  | *ACTBL2* | r | -0.394 | **-0.518** | -0.332 | -0.286 | 0.258 | -0.325 | 0.545 | -0.006 | -0.284 |
|  |  | p | 0.131 | **0.040** | 0.208 | 0.282 | 0.416 | 0.301 | 0.066 | 0.986 | 0.371 |
| **Structure** | *ACTN2* | r | 0.091 | -0.171 | -0.205 | -0.233 | 0.146 | -0.479 | 0.615 | -0.187 | -0.501 |
|  |  | p | 0.737 | 0.527 | 0.445 | 0.384 | 0.648 | 0.114 | 0.033 | 0.603 | 0.096 |
|  | *CAPZB* | r | -0.200 | 0.044 | 0.007 | 0.358 | 0.104 | 0.469 | -0.237 | 0.333 | **0.751** |
|  |  | p | 0.457 | 0.871 | 0.978 | 0.172 | 0.745 | 0.123 | 0.456 | 0.346 | **0.004** |
|  | *CDH15_1* | r | 0.394 | 0.105 | -0.073 | -0.051 | 0.021 | -0.486 | 0.419 | -0.393 | **-0.709** |
|  |  | p | 0.131 | 0.696 | 0.785 | 0.849 | 0.948 | 0.108 | 0.174 | 0.259 | **0.009** |
|  | *CDH15_2* | r | 0.394 | 0.141 | -0.008 | -0.175 | 0.021 | -0.501 | 0.468 | -0.272 | **-0.740** |
|  |  | p | 0.131 | 0.602 | 0.973 | 0.515 | 0.948 | 0.097 | 0.124 | 0.445 | **0.005** |
|  | *CMYA5* | r | 0.038 | -0.158 | -0.152 | -0.227 | 0.202 | -0.143 | 0.489 | -0.212 | -0.568 |
|  |  | p | 0.888 | 0.556 | 0.573 | 0.397 | 0.527 | 0.656 | 0.106 | 0.556 | 0.053 |
|  | *CORO6* | r | 0.311 | 0.085 | -0.032 | -0.239 | -0.069 | -0.542 | 0.328 | -0.333 | **-0.695** |
|  |  | p | 0.239 | 0.753 | 0.904 | 0.372 | 0.829 | 0.068 | 0.296 | 0.346 | **0.012** |
|  | *MYBPC2* | r | -0.279 | 0.141 | 0.217 | 0.115 | -0.013 | 0.444 | -0.377 | 0.309 | **0.684** |
|  |  | p | 0.294 | 0.602 | 0.418 | 0.671 | 0.965 | 0.147 | 0.226 | 0.384 | **0.014** |
|  | *NEB* | r | 0.308 | -0.064 | -0.427 | -0.056 | -0.069 | -0.528 | 0.314 | -0.357 | **-0.618** |
|  |  | p | 0.244 | 0.811 | 0.098 | 0.836 | 0.829 | 0.077 | 0.319 | 0.310 | **0.032** |
|  | *OBSCN_1* | r | -0.091 | 0.252 | 0.303 | 0.376 | -0.237 | 0.245 | -0.524 | 0.212 | 0.494 |
|  |  | p | 0.737 | 0.344 | 0.253 | 0.151 | 0.456 | 0.442 | 0.080 | 0.556 | 0.102 |
|  | *OBSCN_2* | r | -0.188 | 0.017 | -0.134 | 0.363 | -0.335 | 0.182 | **-0.608** | 0.175 | **0.698** |
|  |  | p | 0.485 | 0.948 | 0.619 | 0.166 | 0.286 | 0.571 | **0.035** | 0.627 | **0.011** |
|  | *OBSCN_3* | r | 0.167 | -0.055 | -0.097 | -0.159 | -0.244 | -0.472 | 0.146 | -0.333 | -0.498 |
|  |  | p | 0.534 | 0.837 | 0.719 | 0.555 | 0.443 | 0.121 | 0.648 | 0.346 | 0.099 |
|  | *OBSCN_4* | r | 0.200 | -0.035 | -0.004 | -0.231 | 0,000 | -0.423 | 0.482 | -0.224 | **-0.709** |
|  |  | p | 0.457 | 0.896 | 0.986 | 0.387 | 1,000 | 0.169 | 0.112 | 0.533 | **0.009** |
|  | *TNNT2* | r | 0.179 | -0.044 | -0.004 | -0.253 | -0.027 | -0.318 | 0.363 | -0.163 | **-0.642** |
|  |  | p | 0.506 | 0.871 | 0.986 | 0.342 | 0.931 | 0.312 | 0.245 | 0.651 | **0.024** |
|  | *TNNT3* | r | 0.176 | -0.011 | 0.033 | 0.022 | -0.125 | **-0.616** | 0.293 | **-0.648** | ***-0.839*** |
|  |  | p | 0.513 | 0.965 | 0.901 | 0.935 | 0.696 | **0.032** | 0.354 | **0.042** | ***0.001*** |
|  | *TPM2* | r | **-0.556** | -0.370 | -0.011 | 0.191 | 0.398 | 0.448 | 0.048 | 0.515 | ***0.807*** |
|  |  | p | **0.025** | 0.157 | 0.965 | 0.476 | 0.199 | 0.143 | 0.879 | 0.127 | ***0.001*** |
|  | *TTN_1* | r | 0.061 | -0.279 | -0.339 | -0.268 | 0.286 | -0.151 | **0.650** | -0.115 | -0.470 |
|  |  | p | 0.820 | 0.294 | 0.197 | 0.314 | 0.366 | 0.640 | **0.022** | 0.751 | 0.122 |
|  | *TTN_2* | r | 0.144 | -0.141 | -0.192 | -0.311 | 0.153 | -0.276 | 0.475 | -0.296 | **-0.712** |
|  |  | p | 0.594 | 0.602 | 0.475 | 0.240 | 0.633 | 0.383 | 0.118 | 0.404 | **0.009** |
| **Myokine** | *BDNF_1* | r | 0.385 | 0.014 | -0.363 | -0.185 | 0.091 | 0.073 | 0.405 | -0.236 | -0.340 |
|  |  | p | 0.141 | 0.956 | 0.166 | 0.490 | 0.778 | 0.820 | 0.191 | 0.511 | 0.279 |
|  | *BDNF_2* | r | -0.005 | 0.197 | 0.026 | 0.331 | -0.307 | 0.325 | **-0.664** | 0.103 | **0.684** |
|  |  | p | 0.982 | 0.464 | 0.922 | 0.211 | 0.331 | 0.301 | **0.018** | 0.776 | **0.014** |
|  | *C10orf71* | r | 0.344 | -0.047 | -0.236 | 0.151 | 0.195 | -0.185 | 0.356 | -0.587 | **-0.705** |
|  |  | p | 0.191 | 0.862 | 0.377 | 0.577 | 0.541 | 0.563 | 0.255 | 0.073 | **0.010** |
|  | *CXCL13_1* | r | -0.305 | -0.473 | -0.191 | -0.261 | 0.223 | -0.283 | **0.615** | -0.139 | -0.470 |
|  |  | p | 0.249 | 0.063 | 0.479 | 0.328 | 0.484 | 0.371 | **0.033** | 0.701 | 0.122 |
|  | *CXCL13_2* | r | -0.071 | -0.250 | -0.134 | -0.042 | -0.041 | -0.532 | 0.216 | -0.296 | **-0.589** |
|  |  | p | 0.795 | 0.350 | 0.619 | 0.874 | 0.896 | 0.074 | 0.498 | 0.404 | **0.043** |
|  | *CXCL2* | r | -0.347 | -0.020 | 0.013 | -0.033 | 0.286 | 0.479 | -0.069 | **0.721** | ***0.800*** |
|  |  | p | 0.187 | 0.939 | 0.961 | 0.901 | 0.366 | 0.114 | 0.829 | **0.018** | ***0.001*** |
|  | *FGF21_1* | r | -0.011 | -0.135 | -0.019 | -0.045 | 0.091 | -0.227 | 0.405 | -0.442 | **-0.674** |
|  |  | p | 0.965 | 0.617 | 0.943 | 0.866 | 0.778 | 0.476 | 0.191 | 0.200 | **0.016** |
|  | *FGF21_2* | r | 0.250 | -0.064 | -0.130 | -0.342 | 0.091 | -0.227 | 0.482 | -0.030 | -0.571 |
|  |  | p | 0.350 | 0.811 | 0.631 | 0.194 | 0.778 | 0.476 | 0.112 | 0.933 | 0.052 |
|  | *HGF* | r | 0.438 | 0.023 | -0.394 | -0.082 | -0.076 | -0.189 | 0.272 | -0.418 | -0.494 |
|  |  | p | 0.089 | 0.931 | 0.130 | 0.761 | 0.812 | 0.556 | 0.391 | 0.229 | 0.102 |
|  | *IL13* | r | 0.317 | 0.114 | -0.164 | 0.047 | -0.272 | -0.469 | -0.091 | **-0.648** | **-0.691** |
|  |  | p | 0.231 | 0.672 | 0.543 | 0.862 | 0.391 | 0.123 | 0.778 | **0.042** | **0.012** |
|  | *IL8* | r | -0.202 | **-0.538** | -0.458 | -0.022 | 0.202 | -0.388 | 0.279 | -0.224 | -0.424 |
|  |  | p | 0.451 | **0.031** | 0.074 | 0.935 | 0.527 | 0.211 | 0.378 | 0.533 | 0.168 |
|  | *OSTN* | r | -0.194 | -0.282 | -0.146 | -0.140 | 0.055 | -0.315 | 0.384 | -0.357 | **-0.589** |
|  |  | p | 0.471 | 0.289 | 0.588 | 0.604 | 0.862 | 0.318 | 0.217 | 0.310 | **0.043** |
| **Myogenesis** | *MYOF* | r | -0.026 | 0.194 | 0.251 | 0.333 | -0.286 | 0.269 | **-0.657** | 0.054 | 0.421 |
|  |  | p | 0.922 | 0.471 | 0.347 | 0.206 | 0.366 | 0.396 | **0.020** | 0.881 | 0.172 |
|  | *NCAM1* | r | 0.464 | 0.200 | -0.081 | -0.144 | -0.231 | -0.465 | 0.062 | -0.563 | ***-0.796*** |
|  |  | p | 0.069 | 0.457 | 0.764 | 0.593 | 0.471 | 0.126 | 0.845 | 0.089 | ***0.001*** |
|  | *VCAM1* | r | 0.188 | -0.150 | -0.288 | -0.201 | 0.125 | -0.304 | 0.433 | -0.272 | **-0.754** |
|  |  | p | 0.485 | 0.579 | 0.278 | 0.455 | 0.696 | 0.335 | 0.159 | 0.445 | **0.004** |
|  | *MYH4* | r | -0.011 | -0.194 | -0.140 | -0.154 | 0.181 | -0.245 | 0.496 | -0.309 | **-0.667** |
|  |  | p | 0.965 | 0.471 | 0.603 | 0.566 | 0.571 | 0.442 | 0.101 | 0.384 | **0.017** |
|  | *PAX3* | r | -0.188 | -0.311 | -0.161 | -0.241 | 0.209 | -0.241 | **0.580** | -0.224 | -0.477 |
|  |  | p | 0.485 | 0.239 | 0.551 | 0.369 | 0.512 | 0.449 | **0.047** | 0.533 | 0.116 |

SDS: standard deviation score: BMI: Body mass index.

In bold are highlighted the significant associations with a p-value of 0.05. In grey, significant associations after multiple testing correction (p-value <0.001) are highlighted.
